# Medical student perceptions of their knowledge and skills in pharmacology in their first and final clinical years

**DOI:** 10.15694/mep.2019.000042.1

**Published:** 2019-03-08

**Authors:** Shane Bullock, Anne Leversha

**Affiliations:** 1 School of Rural Health

**Keywords:** Medical education, pharmacology, prescribing, curriculum, teaching and learning.

## Abstract

This article was migrated. The article was marked as recommended.

Medical student perceptions of their knowledge and skills in pharmacology in their first and final clinical years were investigated. An online survey investigated qualitative and quantitative data. Resources that the students relied on to support their knowledge in this area included pharmacists and the Australian Medicines Handbook. Suggested areas for curriculum review include having more formal teaching in this area and having more prescribing practice.

## Introduction

Pharmacology is an increasingly important area of study for medical students because it provides the scientific basis for safe and rational prescribing of medications ( Gwee, 2009) and the foundation for practice ( Richir *et al*., 2008; Lum, Mitchell and Coombes, 2013). However, preparation in pharmacology presents significant challenges to medical students and young doctors, with an increasing number of medications on the market and an elevated risk of medication misadventure, particularly with respect to polypharmacy ( Hubbard *et al.,* 2015; Jokanovic *et al.,* 2015; Scott *et al*., 2015; Ferguson, Keyworth and Tully, 2018).

Safe and effective medication use is a global issue. Educational institutions and professional organisations have important roles to play in meeting the goal recently stated by the World Health Organization (WHO) to reduce the severe avoidable harms related to medications ( WHO, 2017). Furthermore at the national level, there is the National Strategy for Quality Use of Medicines and the National Health Service Standards to make the best possible use of medicines to improve health outcomes for all Australians ( DHA, 2002; ACSQHC, 2017).

Students and junior doctors in Australia and the UK have reported feeling poorly prepared with respect to clinical pharmacology ( DEST 2008; Coombes, Mitchell and Stowasser, 2008). An Australian study published in 2013 identified that the majority of junior residents and registrars in the emergency department (ED) had inadequate knowledge about the medications that were most recently prescribed in ED. In addition, the perceptions of their knowledge of selected common and recently prescribed medications were inflated in relation to their actual knowledge ( Starmer *et al*, 2013). Some students may think that they will improve their prescribing skills *after* finishing medical school, but research shows that despite gains in general experience, prescribing skills do not improve much after graduation ( de Vries *et al.*, 1994). In studies of both final year medical students and interns ( Coombes, Mitchell and Stowasser, 2008; Heaton, Webb and Maxwell, 2008), there was consensus that there was a lack of training and preparedness for safe prescribing.

However, it should be noted that in response to this lack of preparedness there have been some innovative approaches, such as the Prescribing Safety Assessment (PSA) ( Maxwell *et al*, 2017), pharmacist-facilitated teaching ( Leversha and Pedlar, 2008; Maguire *et al*, 2015), and the use of lists of core medicines to familiarise students with commonly used medications ( de Vries *et al.*, 1994; Gaetani and Yeo, 2012).

This study investigates the perceptions of students in their first clinical year and in their final clinical year, with respect to their knowledge and skills in therapeutics, and to aspects of their teaching and learning experience in pharmacology. Our study is distinctive in that usually such studies focus on final year students. In this study, the perceptions of students in their first and final clinical years was investigated. Recommendations regarding enhancing this component of the medical curriculum will be made.

## Methods

Aspects of pharmacology that were investigated were based on previous studies ( Manias and Bullock, 2002; Heaton, Webb and Maxwell, 2008; Naritoku and Faingold, 2009; O’Shaughnessy *et al*., 2010). This helped inform the types of content explored in this study.

A number of elements were ranked on a 6-point likert scale. The elements were rated as follows:

• **Knowledge** was rated ‘poor to excellent’;
• **Managing a patient’s treatment** was rated from ‘not at all confident’ to ‘very confident’;
• **Value of learning activity** was rated from ‘not at all’ to ‘completely’;
• **Clinical resources** was rated from ‘least useful’ to ‘most useful’.

An acceptable rating score was designated as being between 4-6, and an unacceptable rating score was designated as being between 1-3.

The study comprised of a one group post-test design using online surveys to collect both quantitative and qualitative data. In a cross-sectional study, students enrolled in the graduate entry MBBS course that were in their first clinical year and those in their final year of study in the same calendar year were targeted. These two cohorts were invited by email to participate in an online survey regarding their perceptions of the teaching and learning of their pharmacology and therapeutics components of the course. The two surveys did include some of the same questions, however the individual cohort surveys did contain different questions more specific to their year of study. The cohorts were surveyed in the first semester. The same surveys were administered again in the following year to the next intake of students in these year levels of the course to increase the sample size. The collected data was tested for homogeneity and then pooled across the two years.

Quantitative and qualitative survey responses from participants were self-recorded anonymously online using Qualtrics ^TM^ software. Likert scale questions provided the quantitative data, and the qualitative data was obtained from open-ended free text questions. Frequency of Likert-scale responses were calculated and thematic analysis was performed on the open-ended question responses. The responses of the first clinical year and final clinical year students to the knowledge of drug actions, adverse drug reactions, pharmacokinetics and drug interactions were analysed using the Kruskal-Wallis test.

Informed consent was sought from and provided by all participants, with ethics approval for the study granted by the Monash University Human Research Ethics Commitee (CF14/1188 - 2014000529).

## Results/Analysis

The survey response rates for the first clinical year and the final year student groups were 30% and 25.5% respectively.

The two cohorts of students were asked to rate selected aspects of their pharmacology experience so far in their course ( Table 1).

**Table 1.** Ratings of Aspects of Pharmacology (in percentage of responses)

Aspects of Pharmacology	Clinical Year	Rating					
		1 (Poor)	2	3	4	5	6 (Excellent)
Knowledge of ADRs	First Year	0	27.3	45.5	27.3	0	0
	Final Year	3.3	6.7	36.7	36.7	16.7	0
Knowledge of Pharmacokinetics	First Year	0	23.5	50	20.6	5.9	0
	Final Year	13.3	23.3	26.7	20	10	6.7
Knowledge of Drug Interactions	First Year	0	23.3	60	10	6.7	0
	Final Year	10	30	23.3	20	16.7	0
Knowledge of Drug Actions	First Year	0	20.6	17.6	41.2	20.6	0
	Final Year	0	10	6.7	43.3	26.7	13.3
How prepared did you feel for the applied pharmacology program?	First Year	0	21.2	39.4	33.3	6.1	0
Deciding appropriate choice of formulation	First Year	0	36.7	33.3	23.3	6.7	0
Knowledge of drug groupings	First Year	0	6.3	18.8	50	25	0
Rate usefulness of preclinical medication list	First Year	0	6.5	22.6	35.5	35.5	0
Selecting therapeutic drug monitoring (TDM)	Final Year	3.3	3.3	30	26.7	33.3	3.3

Student knowledge of drug actions, adverse drug reactions (ADRs), pharmacokinetics and drug interactions were surveyed at both in the first clinical year and the final year. The Kruskal-Wallis test showed that the distribution of responses between first year clinical and final year students is significantly different for knowledge of ADRs (p=0.011) and drug actions (p=0.023), but not for knowledge of pharmacokinetics (p>0.05) or drug interactions (p>0.05). The proportion of first year clinical students that perceived their knowledge of drug actions acceptable was 61.8%, while 83.3% of final year students considered it acceptable. Knowledge of ADRs was rated as acceptable by 27.3% of first year clinical students, and by 53.4% of final year students. Knowledge of pharmacokinetics was rated as acceptable by 26.7% of first clinical year students and 36.7% of final year students, while knowledge of drug interactions was rated as acceptable by 16.7% of first year clinical students and 36.7% of final year students.

When first year clinical students were asked if they felt prepared for their applied pharmacology program only 39.4% answered acceptable. Seventy-five percent of first year clinical students considered their knowledge of drug groupings acceptable, but only 30% responded positively to whether they could decide the appropriate choice of formulation. When asked to rate the usefulness of the core medications list, 71% responded acceptable.

When final year students were asked if they could select therapeutic drug monitoring (TDM), 63.3% regarded this knowledge as acceptable.

Students in their first clinical year considered the most valuable learning environments in order were at the bedside (42.9%), tutorials and self-directed learning (34.3%) and then problem-based learning classes (17.1%). In decreasing order their most preferred to least-preferred form of teaching is lectures, expect-guided problem-solving, tutorial discussions and working through a series of questions.

In free-text commentary, the first clinical year indicated that they wanted to know more about specific drugs, ADRs, drug interactions and antibiotics (choice and dosage). They desired more clinically relevant pharmacology knowledge, more guidance as to the important aspects of pharmacology to know and more ward time with respect to medication usage.

The final year students were asked to indicate their confidence to manage a patient’s treatment using a particular medication or medication group. The ratings are shown in Table 2. An acceptable level of confidence is highest for antihypertensives and narcotic analgesics, and is lowest for the anticonvulsants.

**Table 2.** Confidence of Final Year Students to Manage a Patient’s Treatment with these Medications (in percentage of responses)

Medication or Medication Group	Ratings					
	1 (Not at all confident)	2	3	4	5	6 (Very confident)
Antihypertensives	0	0	10	16.7	53.3	20
Antibiotics	0	6.7	16.7	23.3	43.3	10
Oral hypoglycaemics	0	0	23.3	26.7	33.3	16.7
Warfarin	3.3	0	30	23.3	40	3.3
Antidepressants	0	10	23.3	33.3	20	13.3
Narcotic analgesics	6.7	0	10	40	36.7	6.7
Anticonvulsants	20	23.3	40	16.7	0	0
Heparin	10	20	30	23.3	16.7	0
Disease modifying antirheumatic drugs (DMARDs)	23.3	33.3	23.3	6.7	10	3.3

Final year respondents rated a number of resources on a scale of least useful to most useful to them in that year of study. The proportion rated most useful for each resource was pharmacists (63.3%), the Australian Medicines Handbook [AMH] (33.3%), registrars (23.3%), consultants (13.3%), the MIMS clinical resource (13.3%) student peers (3.3%) and interns (3.3%).

Eighty-three percent of final year respondents indicated that they would have liked more practice in prescribing. They acknowledged that while there are legal constraints prohibiting them from writing actual patient prescriptions, this could be achieved in online tutorials or supervised practical-based classes.

Generally, final year students perceived their pharmacology experiences to be challenging in nature and indicated that there should be more formal systematic teaching of pharmacology in the clinical years. These views are reflected by the following student comments:

“It was initially overwhelming but the more exposure to it the more comfortable I got with it. It would be good to have more formal teaching around pharmacology.”

“I feel very underconfident, but I’ve been told it’ll get better with experience”

“Prescribing medications is the area I feel most unprepared for when entering my intern year”

“I feel that this is a neglected area of the course that is not taught in a systematic way. It’s currently taught haphazardly and your experience depends largely on what clinical teachers you are exposed to. Weekly tutorials devoted just to prescribing would be the best way to fix this, I think.”

## Discussion

When the distribution of responses for each of the four selected aspects of pharmacology were compared in first year and final year clinical students, the responses for knowledge of ADRs and drug action were significantly different across the two clinical years. The responses for the final clinical year were skewed toward higher ratings. This suggests that across the clinical years the students perceive their knowledge of these aspects improving, which is reassuring.

Such a difference between the distribution of responses of the two clinical years was not demonstrated for pharmacokinetics and drug interactions. We suggest an improvement in knowledge in these aspects is not perceived. A sound knowledge of pharmacokinetics and drug interactions is an important component of patient safety and care ( Coombes, Mitchell and Stowasser *,* 2008, Lum, Mitchell and Coombes, 2013), and warrants more attention within curricula design delivery. Individual confidence in that knowledge cannot be underestimated. Pharmacy students have been shown to have significantly better knowledge of drug interactions compared to medical students ( Harrington *et al*., 2011; Keijsers *et al*., 2014; Hilmer, Seale and Carroll, 2014) which suggests that this may be due to differences in undergraduate education between these two health professions. Medical students in our study indicated that they want more formal teaching in pharmacology in the clinical years. Improved opportunities for integrated and applied structured learning in the clinical years of these aspects may be helpful. This study provides further support for pharmacist-facilitated pharmacology learning ( Maguire *et al.,* 2015).

In our study, 63.3% of final year students rated their knowledge of therapeutic drug monitoring (TDM) as being acceptable. In a previous study medical students have acknowledged that ADR reporting improves drug safety and patient care ( Shankar *et al.,* 2011). Effective TDM and ADR reporting draws strongly on well developed clinical skills of observation and recognising variation from normal. Students may be confident and highly motivated in this aspect of pharmacology. A good workable knowledge of drug actions would be expected to enhance recognition of possible ADRs.

The first year clinical students identified their most valuable learning environment as the bedside; but interestingly, lectures were the most highly valued teaching activity, whereas the problem-based learning is the least valued. The students in these cohorts have shown a preference for a combination of experiential and didactic learning.

Johannsen and colleagues found that across their training medical students had more confidence in their declarative pharmacology knowledge (i.e. indications of drugs) compared to application-oriented knowledge (i.e. applying a drug treatment) ( Johannsen *et al*., 2018). In our study we found that the confidence of final year students to manage a patient’s treatment with selected medications is not actually equivalent across all drug classes. There are some drug classes that they feel confident with, such as antihypertensive agents and narcotic analgesics, while for other classes they have very low levels of confidence, such as in the case of heparin and anticonvulsants. Reasons for these differences may reflect the frequency of exposure to these medication classes as a student in clinical placement. Antihypertensives and narcotic analgesics are amongst the most widely prescribed medications. The complexity of the management of therapy in relation to epilepsy and the use of heparin may also be a factor affecting confidence. Medical curricula must take such differences into account in the delivery of drug class knowledge.

It is significant that final year medical students recognise the expertise of other health professionals, particularly pharmacists, in medication decisions. This is worthy of further attention within program delivery. Less surprising is the recognition and usage of important clinical resources such as the AMH.

Final year students strongly desire more prescribing practice. This finding is consistent with the literature regarding medical graduates entering internship ( Coombes, Mitchell and Stowasser, 2008; Hilmer *et al*., 2009; Cameron *et al*., 2014). In response to this need, our University has adopted and adapted the PSA ( Maxwell *et al*., 2017) as a formative activity in the final clinical year, as have other Australian and New Zealand medical schools.

When final year students were asked the greatest concern with respect to their prescribing role as an intern the most common response related to knowing the correct dose and drug interactions. The dose of medications has been seen to be in the realm of the intern and this aspect of knowledge does not appear to be emphasised in pre-registration medical courses. However, there is a place for an understanding of the dosages of commonly used medications. Without considering this aspect of medication use, dose-related issues may be ignored. Curriculum developers should consider giving more attention to this area in the course.

Medical students highly valued their pharmacology education, particularly the formal teaching in pre-clinical year. There is a desire for more formal teaching of pharmacology and therapeutics in the clinical years. Given that the timing of delivery of formal teaching in pharmacology in medical courses across Australia occurs in the early years ( Lloyd *et al.*, 2013), this same desire would be expected in Australian medical students in universities other than ours. There is already ad hoc bedside teaching occurring with respect to medications. However, this needs to be supported by structured programs, which are well mapped to the overall curricula. There are excellent resources also currently available in Australia to support this learning, such as the National Prescribing Service MedicineWise National Prescribing Curriculum, to which students should be directed. However, students want more practice in considering doses, drug interactions, ADR’s, case based scenarios and prescription writing.

In the WHO Guide to Good Prescribing it was noted that although different approaches are taken in education, such as copying the prescribing behaviour of clinical teachers and using drug-centred or disease-centred references, the acquisition of prescribing skills remains weak ( de Vries *et al.*, 1994). The issues that have been identified by the students in this study as important components of medication management and patient safety warrant due consideration in the curriculum.

### Study Limitations

The study comprises a small number of student respondents within one Australian medical school. Therefore, the conclusions need to be viewed in this context. It is a cross-sectional rather than longitudinal study, so individual student responses across the two clinical years cannot be paired. Focus group interviews would have been useful to further explore student responses.

## Conclusion

The study investigated the perceived level of preparedness of first year clinical and final year clinical medical students with respect to their knowledge and skills in clinical pharmacology and therapeutics. It highlighted perceived strengths and weaknesses in knowledge, suggested areas for curriculum review and identified the resources that the students relied on to support their knowledge in this area.

## Take Home Messages

• Medical students in this study want more formal pharmacology and therapeutics teaching across their course
• Medical students in this study want more prescribing practice in the final year of their course
• Pharmacist-facilitated learning in pharmacology and therapeutics is greatly valued by medical students and these opportunities should be considered in the delivery of programs

## Notes On Contributors

Shane Bullock has been involved in the education of student health professionals and scientists for more than 30 years. Shane has co-authored three Australian textbooks, *Fundamentals of Pharmacology*, *Psychopharmacology for Health Professionals* and *Principles of Pathophysiology.* He has published in health professional education, in particular with respect to pharmacology knowledge.

Anne Leversha is a Senior Lecturer at Monash University in the Faculty of Medicine, Nursing and Health Sciences and the Faculty of Pharmacy and Pharmaceutical Sciences. Anne is a clinical pharmacist and is a Fellow of The Society of Hospital Pharmacists of Australia (SHPA).
