# Supplementary material for: Medical student perceptions of their knowledge and skills in pharmacology in their first and final clinical years
Source: MedEdPublish (2016). 2019 Mar 8;8:42. [Version 1] doi: 10.15694/mep.2019.000042.1 (PMC10712612; doi:10.15694/mep.2019.000042.1)
Supplement: Peer review history

# Reviewer report: Reviewer response for version 1

**DOI:** 10.21956/mep.19767.r29683

**Published:** 2019-03-09

**Authors:** BALAJI ARUMUGAM

**Affiliations:** 1 TAGORE MEDICAL COLLEGE AND HOSPITAL

This review has been migrated. The reviewer awarded 4 stars out of 5 Thanks for the invite. The online survey on first and final year stuents on pharmacology and its application knowledge was well studied using qualitative and quantitative data, compared using the valid statistical test. The data from final year students in managing patients treatment with the rating scale was excellently presented in this study.The study investigated the perceived level of preparedness of first year clinical and final year clinical medical students with respect to their knowledge and skills in clinical pharmacology and therapeutics. I wonder why the first year students were involved in this study. !!!!Similarly the study would have yielded better results if two groups like final year and interns were compared rather than first year and final year students. Nevertheless its a good study for the people in the same field.

Reviewer Expertise:

NA

No decision status is available

# Reviewer report: Reviewer response for version 1

**DOI:** 10.21956/mep.19767.r29684

**Published:** 2019-03-08

**Authors:** P Ravi Shankar

**Affiliations:** 1 American International Medical University

This review has been migrated. The reviewer awarded 4 stars out of 5 Thank you for the invitation to review this interesting manuscript. The authors have compared medical students’ perception regarding their knowledge and skills about pharmacology among first and final year students at an Australian University. I have been teaching pharmacology for nearly twenty years and am of the opinion that the prescribing skills of medical students need to be strengthened. It has been erroneously assumed that once a correct diagnosis has been made the correct treatment will automatically follow. Prescribing is a complex task which requires consideration of a variety of factors. The prescribing skills assessment (PSA) is a good initiative toward promoting safe use of medicines in patients. The number of students who participated in the study has not been mentioned. The response rate was low as is often the case with online surveys. Students wanted sessions on prescribing and greater teaching of pharmacology during the clinical years. Australia has created a number of resources to promote rational and safe use of medicines as mentioned by the authors. Appreciation of the important role of pharmacists as resource persons is to be welcomed. This study will be of special interest to all teachers of pharmacology in medical and health professions schools.

Reviewer Expertise:

NA

No decision status is available
